# Preparation of periodic surface structures on doped poly(methyl metacrylate) films by irradiation with KrF excimer laser

**DOI:** 10.1186/1556-276X-9-591

**Published:** 2014-10-28

**Authors:** Yevgeniya Kalachyova, Oleksiy Lyutakov, Petr Slepicka, Roman Elashnikov, Vaclav Svorcik

**Affiliations:** 1 Department of Solid State Engineering, Institute of Chemical Technology, Prague 166 28, Czech Republic

**Keywords:** Polymer, Laser, Modification, Surface, Grating

## Abstract

In this work, we describe laser modification of poly(methyl methacrylate) films doped with Fast Red ITR, followed by dopant exclusion from the bulk polymer. By this procedure, the polymer can be modified under extremely mild conditions. Creation of surface ordered structure was observed already after application of 15 pulses and 12 mJ cm^−2^ fluence. Formation of grating begins in the hottest places and tends to form concentric semi-circles around them. The mechanism of surface ordered structure formation is attributed to polymer ablation, which is more pronounced in the place of higher light intensity. The smoothness of the underlying substrate plays a key role in the quality of surface ordered structure. Most regular grating structures were obtained on polymer films deposited on atomically ‘flat’ Si substrates. After laser patterning, the dopant was removed from the polymer by soaking the film in methanol.

## Background

Illumination of polymers by polarized UV laser beam under specific conditions can induce formation of surface ordered structures [1-6]. Properties and mechanism of grating formation have been extensively studied after their first observation [7]. Now, it is apparent that periodical structures develop on polymeric material surface as a result of the interference between attenuated and diffracted beams [8]. Creation of surface ordered structures occurs only if a threshold value of laser energy is exceeded. Periodicity and amplitude of surface structures depend on applied laser wavelength, polymer's properties, and angle of incidence of the laser beam [9-16]. Grating creation may open new possibilities in a wide range of applications, including microfabrication, surface modifications, and medical applications fields [17-20].

Sufficient amount of absorbed laser energy is necessary to induce a polymer mass redistribution or ablation and creation of periodical structures at polymer surface. Polymers which are not absorbing at the wavelength of laser irradiation may be sensitized by doping with suitable compounds [21,22]. Polymer doping may lower the threshold of ablation and therefore the costs of machining. Broad spectrum of periodic structures may be prepared by properly choosing the polymer and dopant from many possible combinations and by adjusting experimental conditions [23-26].

Poly(methyl methacrylate) (PMMA) is a common material with excellent optical and electrical properties. PMMA weakly absorbs at 248 nm, and the first excimer modification of this polymer, carried out by Kawamura et al. in 1982, was performed on PMMA doped with benzoin [27]. Later, significant attention was paid onto ablation of both pristine PMMA [28-32] and PMMA doped with different compounds [33-37]. Wochnowski et al. [38] and Scully et al. [39] investigated the modification mechanism of PMMA induced by excimer laser irradiation at different wavelengths. The threshold value of the laser energy for the creation of periodical structure on pristine PMMA surface was found to be 400 mJ cm^−2^ (at 248 nm). Srinivasan et al. [40] reported the possibility of decreasing this threshold to 90 mJ cm^−2^ by addition of 2 wt.% of acridine. However, compared to other common polymers (e.g., polystyrene, polyethylene, polyethylene terephthalate), the threshold energy needed for grating formation on PMMA is significantly higher.

Despite a lot of works regarding the excimer modification of PMMA [33-40], several key issues remain unclear: (i) coalescence of grating features, which occurs under higher dose of illumination and leads to abnormal dependence of structure parameters on experimental conditions; (ii) influence of the underlying substrate; and (iii) the possible removal of the dopant after PMMA modification.

Based on the available information, we firstly introduced the method of ripple structure preparation on pristine PMMA at extremely ‘mild’ conditions (for common polymers). Decreasing the threshold value was carried out through the addition of a dopant, followed by laser illumination and dopant removal. We describe the effect of KrF laser irradiation of the doped PMMA in dependence on the substrate's roughness. We also investigated the dynamic of the surface structure formation and prepared a detailed report about the dependence of structure parameters on the condition of both laser treatment and properties of pristine polymer films.

In this work, a new method of grating formation on PMMA films deposited on a carrier substrate at extremely mild conditions is described. The threshold value of the laser energy is lowered by PMMA doping with 2-methoxy-5-(diethylaminosulfonyl)aniline, better known as Fast Red ITR (FR). The surface ordered structures are created by irradiation with KrF laser light, and the dopant is removed from the PMMA bulk after irradiation. The dynamic of the grating formation is described, and the dependence of laser irradiation effects on the smoothness of the carrier substrate is investigated.

## Methods

### Materials

**Figure 1:**
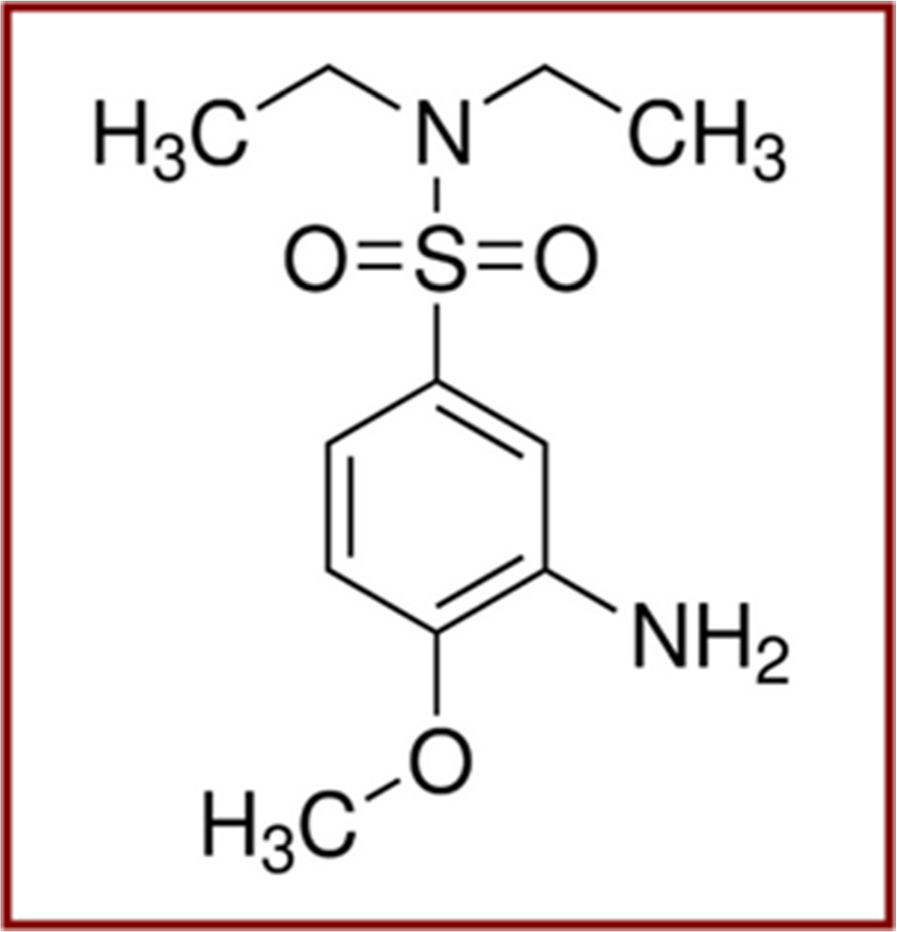
**Chemical structure of Fast Red ITR (FR, absorption at *λ***_**max **_**= 226 nm).**

PMMA of optical purity was supplied by Goodfellow Inc. (Llangollen, UK) and Fast Red ITR (chemical structure is depicted in Figure 1) of 96% grade (*λ*^abs^_max_ = 226 nm) was purchased from Sigma-Aldrich Corp. (St. Louis, MO, USA). Both materials were used without additional purification.

### Sample preparation

The Fast Red-doped PMMA films were prepared by separately dissolving the PMMA (M_w_ ~ 1,500 K) and the Fast Red ITR in 1,2-dichloroethane. Then, 7.0 wt.% PMMA and 2.8 wt.% FR solutions were mixed and spin-coated onto freshly cleaned silicon wafers (crystallographic orientation (100), resistance 0.002 Ω cm, refractive index *n* = 3.50) or glass substrates (supplied by Glassbel Ltd., Prague, Czech Republic). The prepared samples were dried under ambient conditions for 24 h. The thicknesses of the polymer films were measured by profilometry.

### Polymer modification

The samples were irradiated with KrF excimer laser pulses (40 ns, *λ* = 248 nm) at a repetition rate of 10 Hz (Lambda Physik COMPexPro 50, Coherent Inc., Göttingen, Germany). The laser beam was polarized linearly with a cube of UV-grade fused silica with an active polarization layer. According to the preliminary experiments, it was decided to apply several levels of the irradiation energy, relating to different numbers of pulses and fluencies. The samples were irradiated by 1 to 350 laser pulses with laser fluencies from 7 to 13.5 mJ cm^−2^. The samples were mounted onto a translation stage, and different angles (from 0° to 67°) between the sample surface normal and the laser beam were chosen.

### Diagnostic methods

The surface morphology of PMMA and the grating structures was examined with an AFM technique using a VEECO CP II device (‘tapping’ mode, probe RTESPA-CP, spring constant 50 N·m^−1^, Veeco Instruments Inc., Plainview, NY, USA). The mass loss of the samples was measured using a Mettler-Toledo UMX2 microbalance (Mettler-Toledo, Inc., Greifensee, Switzerland). UV/vis spectra were measured using a UV/vis spectrometer Lambda 25 (PerkinElmer, Inc., Waltham, MA, USA).

## Results and discussion

PMMA is known to have weak absorption peak at 248 nm [41,42]. Therefore, the modification of PMMA can be performed by application of ‘high’ laser fluence which can be reduced by adding a dopant with high extinction coefficient. In our case, we used FR as the dopant (chemical structure of FR is presented in Figure 1). FR is well compatible with PMMA. The prepared films are homogeneous, and no dopant aggregation on the polymer surface or in the bulk is observed. The FR compound exhibits strong absorption at 248 nm and shifts optical properties of PMMA into the desired range.

**Figure 2:**
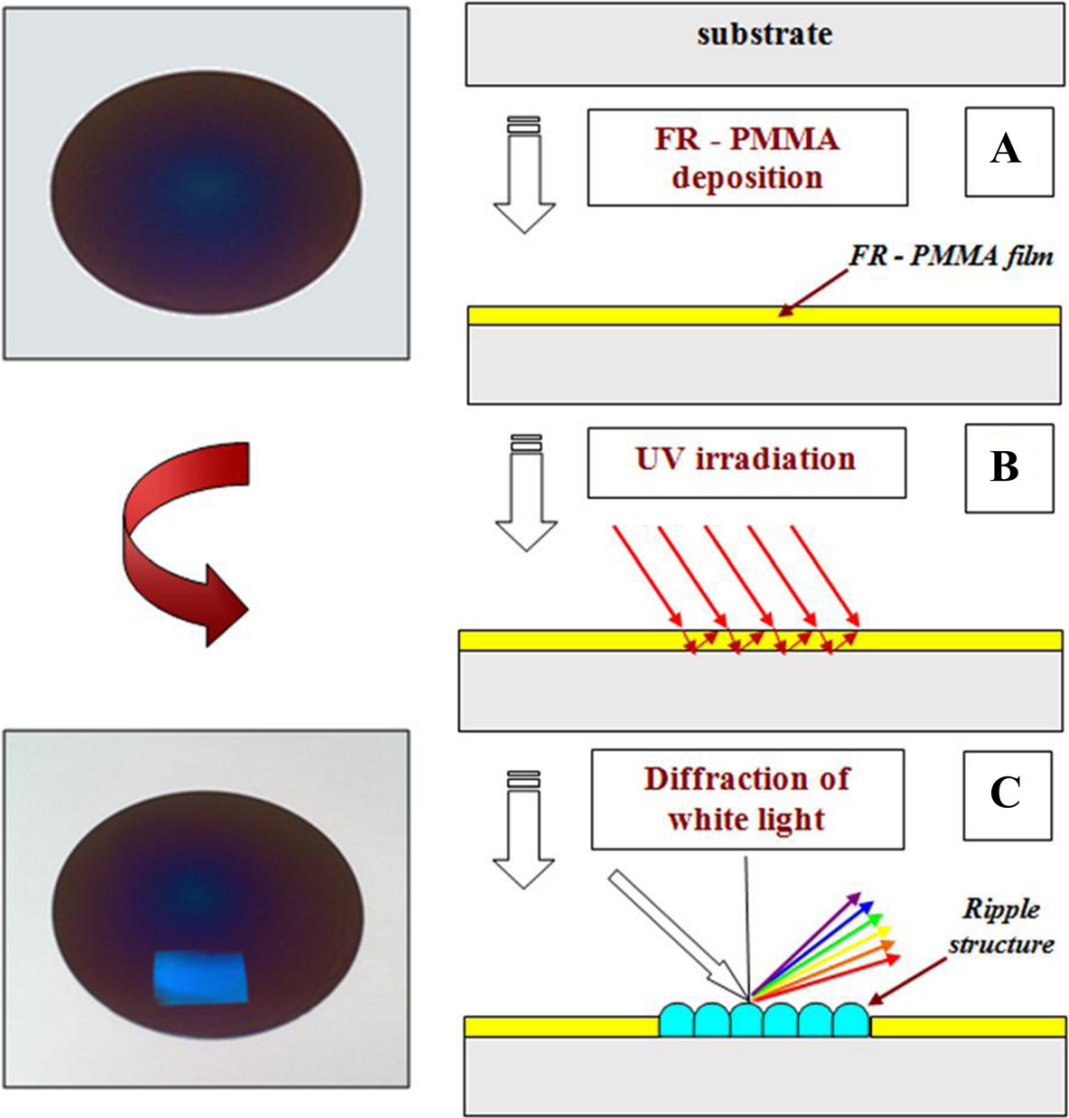
**Scheme of the preparation of surface ordered structures. (A)** PMMA-FR deposition, **(B)** UV laser irradiation, and **(C)** diffraction of white light on prepared periodical structure (on ripples).

After deposition, the PMMA-FR films were irradiated with KrF excimer laser beam (*λ* = 248 nm) under various angles with respect to film surface normal. This process is schematically depicted in Figure 2. At the surface of the thin polymer film, an interference between incident and reflected laser beam occurs which results in spatial redistribution of laser intensity along the sample surface [11]. As a result, surface ordered structures are formed. Diffraction under irradiation with white light (angle-dependent coloring of patterned area) indicates the quality of the created pattern. The left side of Figure 2 gives the sample photo before and after laser modification. The patterned area is clearly visible on the sample. Homogeneous distribution of diffracted light indicates regular periodicity and amplitude of prepared grating.

There are two possible mechanisms of polymer patterning under excimer laser irradiation. The first mechanism consists in polymer mass redistribution, and the second one is related to polymer ablation [11]. To determine the patterning mechanism, we performed gravimetry tests on the samples modified under different angles of incident laser beam. The results are given in Table 1. In all cases, the decrease of the sample mass after laser illumination was observed. This decrease corresponds with the amplitude of the prepared surface ordered structures, i.e., a large mass change was observed for greater amplitude. However, the weight loss-amplitude relationship is not strictly proportional. Additionally, simple calculation shows that the mass loss is at least 1 order of magnitude greater than can be expected if the polymer layer of 30 nm is removed. So, we propose simultaneous but inhomogeneous ablation of the polymer along the sample surface. More pronounced ablation occurs at places of light interference maxima and less pronounced at places of interference minima. As a result, the grating is formed, but it is located on the ground compared to the incident plane of the polymer film. This phenomenon was also verified by profilometry, which indicated loss of several hundred nanometers in film thickness after laser modification.

**Table 1:** Dependences of weight loss and the gratings amplitude on the angle of incidence of laser beam

**Laser irradiation angle (°)**	**∆ m (μg)**	**A (nm)**
0	114 ± 7.2	31
20	97 ± 8.6	29
40	43 ± 5.5	27
50	16 ± 6.5	24

**Figure 3:**
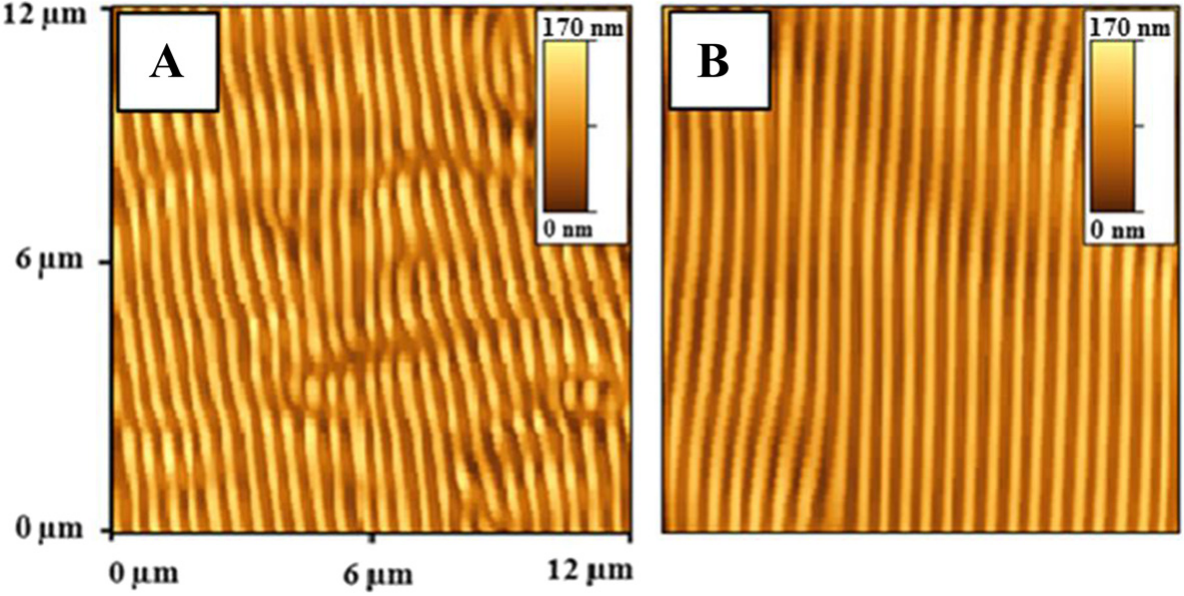
**AFM images of patterned PMMA-FR surface.** Doped polymer films were deposited onto glass **(A)** and atomically flat silicon substrates **(B)**. Insets show the amplitude scale bars.

**Figure 4:**
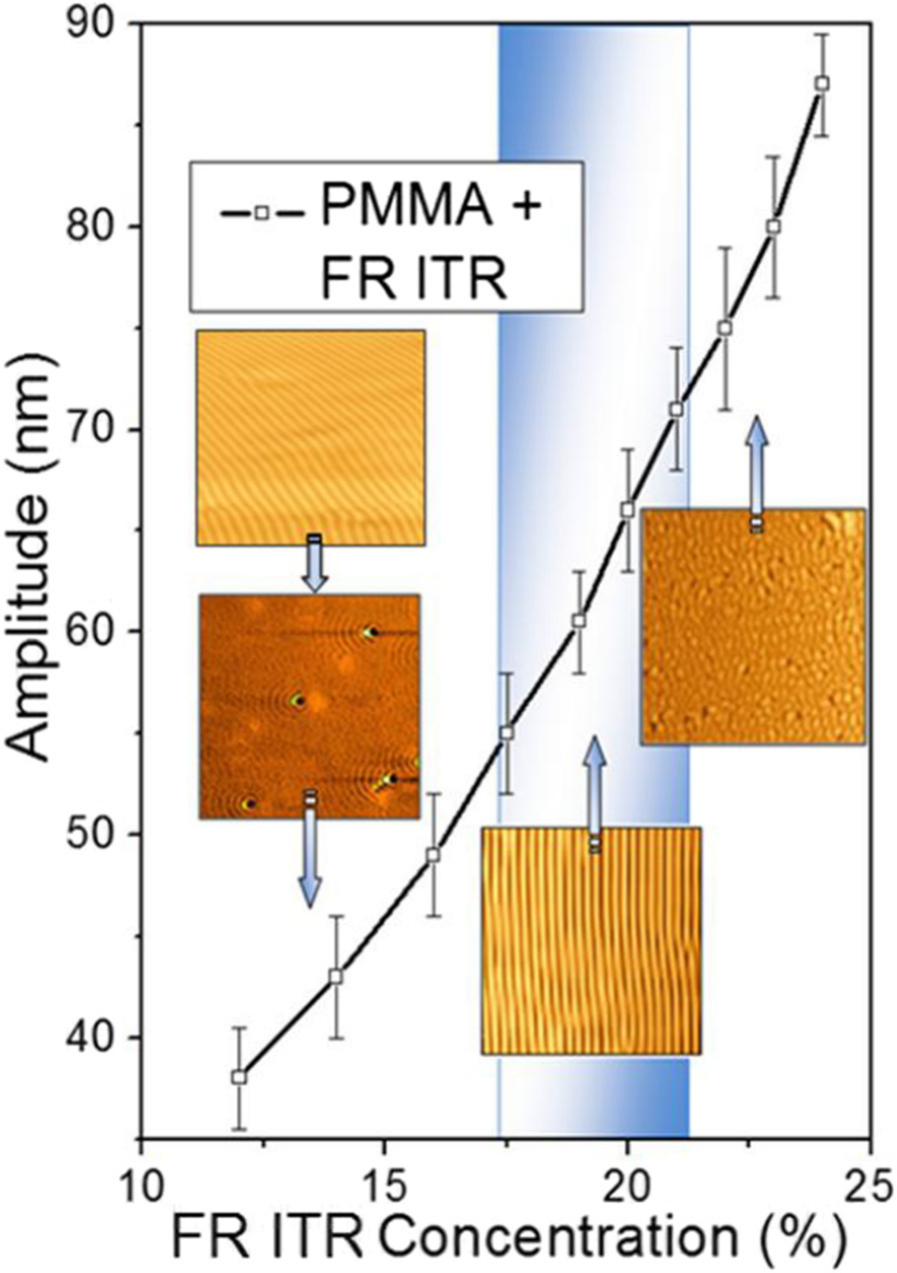
**Dependence of grating amplitude on the concentration of FR.** Laser treatment with 50 pulses and 12 mJ cm^−2^. Insets are AFM images.

The quality of the surface ordered structures prepared by laser beam interference depends strongly on the regularity of interference pattern. In our case, the interference occurs between the incident laser beam and the beam reflected from the underlying substrate. Th properties of incident beam are affected by laser source and optical path (beam collimator, polarizer, presence of impurities, and so on). Reflection from the substrate may introduce additional deviation from ideally collimated and polarized laser beam. This can occur because of different inhomogeneities on the substrate surface, which can scatter the laser beam or change the plane of laser beam polarization. To examine the effect of the underlying substrate, we compare gratings prepared under the same experimental conditions on PMMA-FR films deposited onto microscopic glass and extremely ‘flat’ monocrystalline silicon. Silicon oxide surface layer, typical for Si substrate, was removed by HF etching immediately before polymer deposition. The results of this experiment are shown in Figure 3. It is seen that the quality of grating created on the PMMA-FR/Si substrate is much higher. In case of PMMA-FR/glass, the surface structures consist of several domains, with variable mutual orientation. Grating features are often disrupted or the coalescence with each other is observed. This picture is typical for common case of grating preparation by excimer laser ablation. In the case of PMMA-FR/Si sample, the prepared grating also exhibits some deviation from the ‘ideal’ case, but these deviations are negligible compared with those in the previous case. It appears that the homogeneity of the carrier substrate is critical for obtaining close-to-ideal surface pattern by laser light diffraction. Difference in the refraction indexes of glass and polymer or silicon and polymer must also be taken into account. Glass and polymer have similar refractive indices (1.51 and 1.49, respectively) [43,44]. On the other hand, the difference between polymer and silicon is sufficiently greater (1.49 for polymer and more than 3.5 for Si) [44]. According to Fresnel rules, the amount of reflected energy on polymer/silicon surface is sufficiently higher than that on the polymer/glass surface. Strong reflection can better conserve the laser beam shape, coherence, and light phase shift. Additionally, substrate/polymer reflection will affect the amplitude of interference pattern on polymer surface. So, the difference in grating regularity can be attributed to both parameters - smoothness of substrate's surface and substrate's reflectivity. All further prepared samples were deposited onto silicon substrates.In the next step, we performed a systematic investigation of the parameters of the prepared gratings and their quality which depend on the experimental conditions. At first, we investigated the dependence of the grating amplitude on the amount of added FR which is shown in Figure 4 together with AFM images illustrating the pattern quality. As can be expected, the increase of FR concentration leads to higher energy absorption and amplitude increase. However, not all structures were as regular as that shown in Figure 3B. A too high amount of FR leads to structure disruption and coalescence of grating features (see Figure 4 insets). A too low concentration leads to the structure creation only at several ‘hot’ places randomly distributed on polymer surface. The origin of these hot places can be attributed to inhomogeneities of the polymer film, random deviation in FR concentration, or variations in space distribution of laser beam energy. The range of optimal FR concentration, in which prepared gratings were ‘more’ regular, is depicted in the graph by a shaded area. Some qualitative conclusions about surface structure formation can be made, namely creation of regular grating begins at hot places and tends to form concentric semi-circles with the center at the initial hot place. When the number of hot places is high, they are located closely to each other and created structures overlap during further stages of formation. As a result, a grating pattern is formed. When the number of hot places is small, structure creation occurs only at separate local points. Increasing the laser fluence leads to rather curved geometry of the prepared surface structures, as depicted in Figure 4 insets.

**Figure 5:**
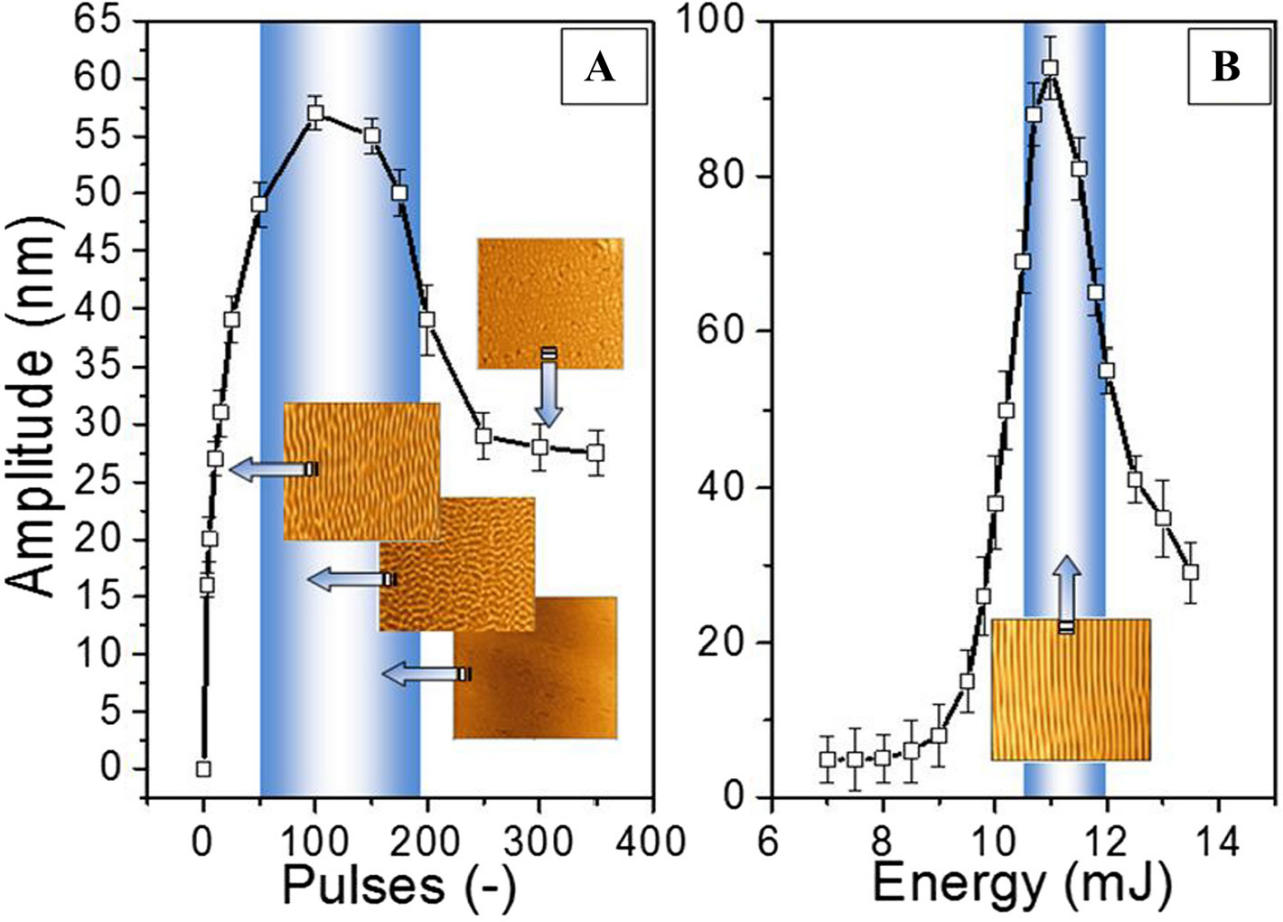
**Dependence of grating amplitude on the number of laser pulses (A) and fluence (B).** Insets are AFM images.

Dependence of grating amplitude on applied energy and number of pulses are shown in Figure 5. As in the previous case, the shaded areas indicate optimal parameters for grating creation. Laser fluence of 12 mJ cm^−2^ was found to be optimal. The optimal number of pulses was found to be very small compared with traditional surface ordered structures creation on common polymers. The number of pulses applied for grating creation is several hundreds or even thousands per sample in common cases. In our case, the initial formation of structure occurs already after 10 pulses and optimal grating was achieved after 50 pulses. The proposed method seems to be unique in terms of the energy and time used - the creation of large-area pattern is performed during several seconds at extremely low-energy costs.

**Figure 6:**
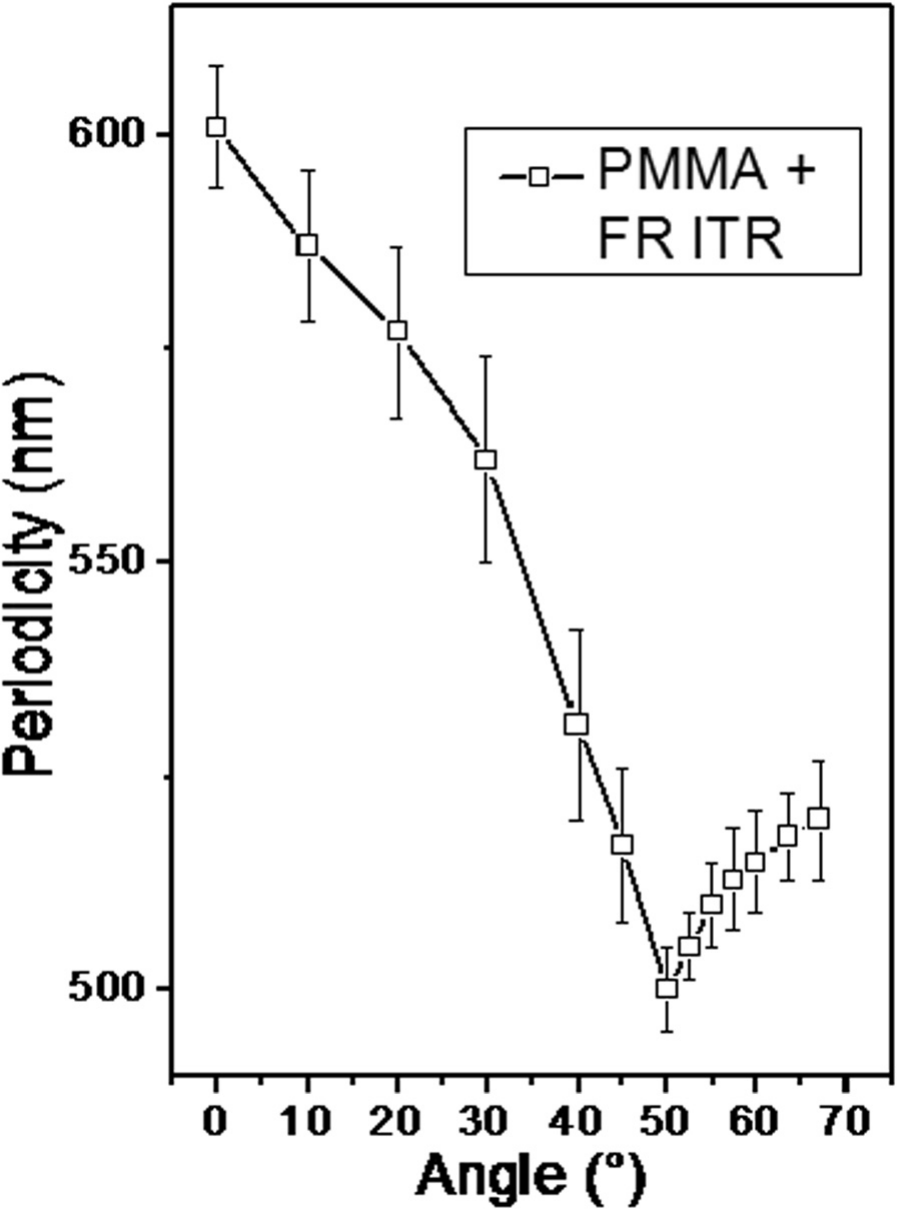
**Dependence of grating periodicity on the angle of incidence of laser beam measured from surface normal.** Laser treatment with 50 pulses and 12 mJ cm^−2^.

**Figure 7:**
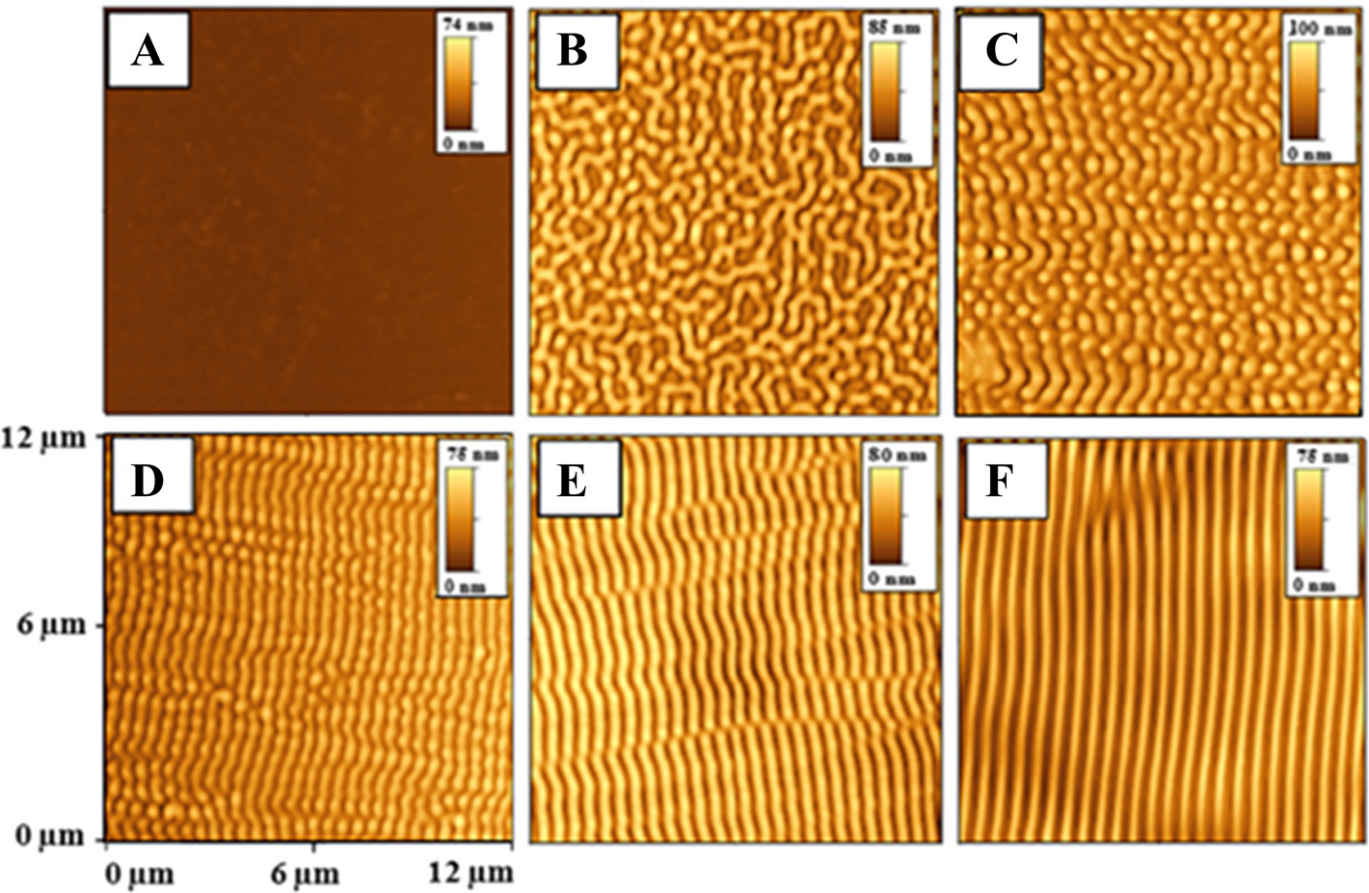
**AFM images of PMMA-FR samples modified under different angles of laser beam incidence.** Laser (50 pulses and 12 mJ cm^−2^). **(A)** Non-irradiated. Irradiated at **(B)** 0°, **(C)** 20°, **(D)** 30°, **(E)** 40°, **(F)** 50°. Insets give the amplitude scale bars.

**Figure 8:**
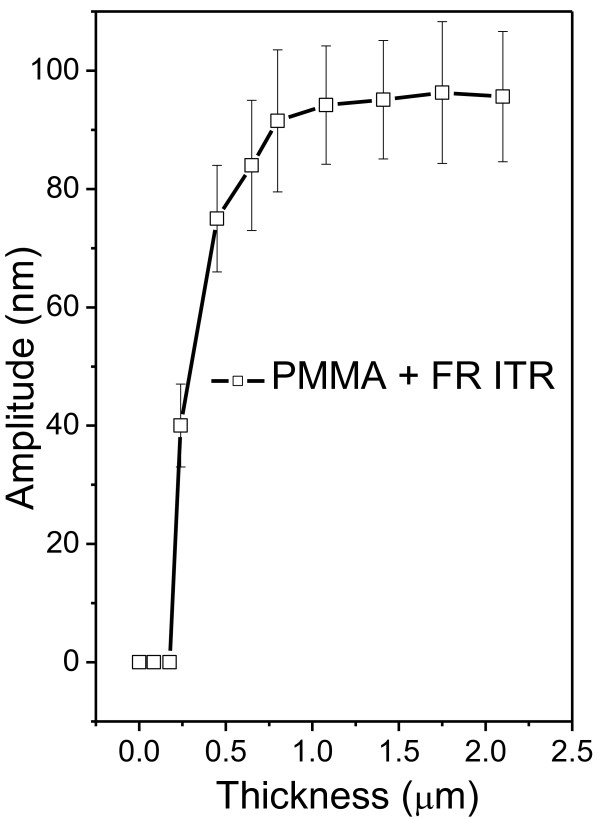
**Dependence of grating amplitude on the initial thickness of PMMA-FR films irradiated by laser.** The laser has 50 pulses and 12 mJ cm^−2^; the concentration of FR was 18%.

**Figure 9:**
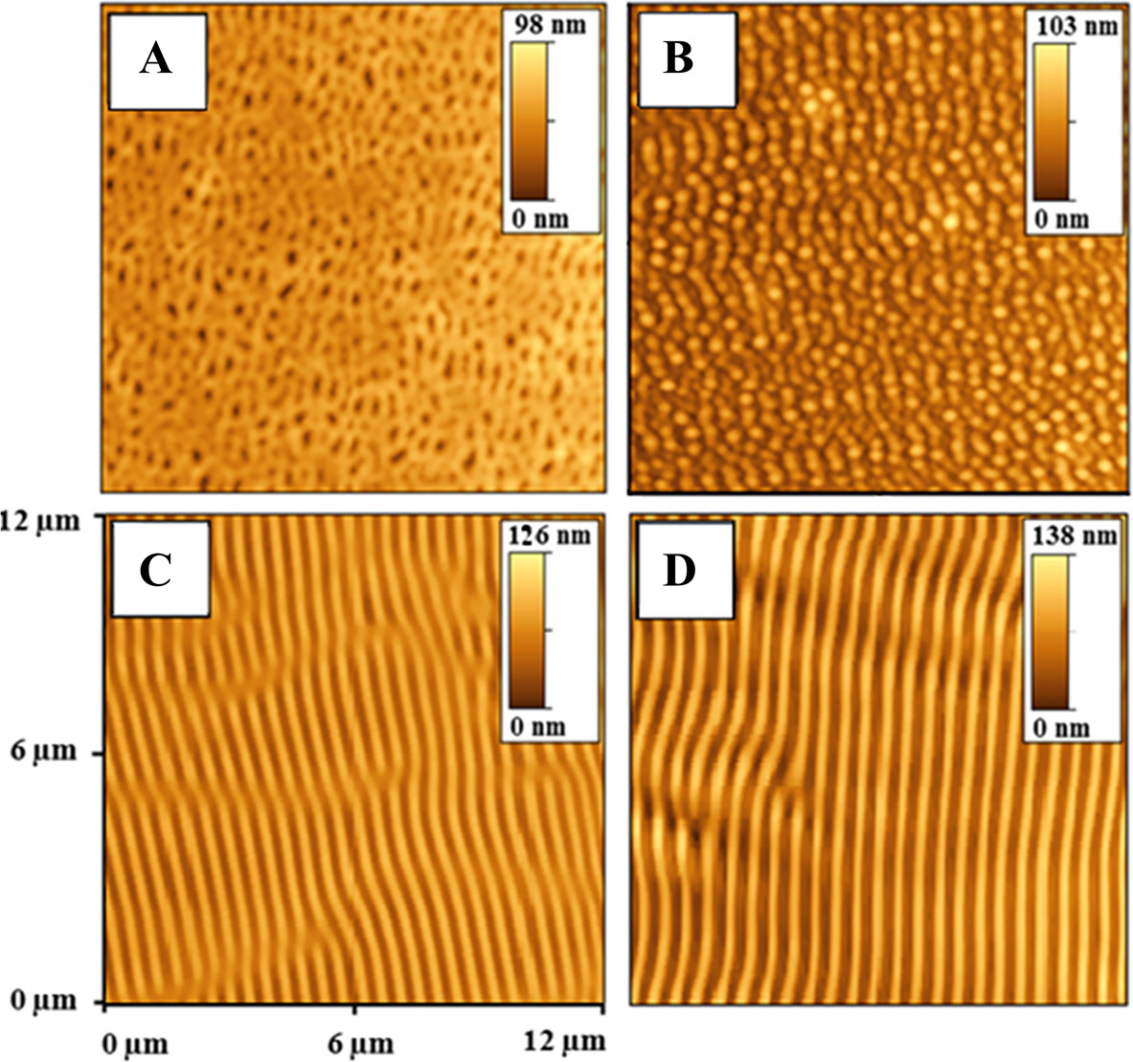
**AFM images of the PMMA-FR films modified by laser (by 50 pulses and 12 mJ cm**^**−2**^**).** Thicknesses of films: **(A)** 0.17 μm, **(B)** 0.2 μm, **(C)** 0.67 μm, **(D)** 2.1 μm.

The dependence of grating periodicity on incidence angle taken from the surface normal is shown in Figure 6. Two distinct regions are apparent from the graph. The region above 50° corresponds to the formation of more regular surface pattern. For angles below 50°, formation of irregular, partially destroyed quasi periodical pattern was observed. The deviation from the proposed behavior may be due to surface structure coalescence and structure degradation. For the sake of clarity, we also show several AFM scans measured on samples modified under different angles (see Figure 7). The modification under angles close to surface normal leads to surface structure in the form of a disrupted structure. If the angle of incidence increases, the structures become more regular. At 50°, the prepared structure is highly ordered and seems to be closed to ‘ideal’. One of the possible explanations consists in changes of laser energy distributions. When the angle of the laser beam incidence is close to surface normal, significant part of light energy penetrates into the polymer film, reflects from silicon substrate, and comes back to interact with the incident light. In this case, amplitude of interference pattern is pronounced, but degradation of polymer films occurs. When the angle of the laser beam incidence is moving away from the surface normal, the amount of penetrated light decreases and grating becomes more regular. When the certain value of ‘optimal’ angle is achieved, high regular grating with approximately 500 nm periodicity is produced (Figures 6 and 7). Another possible explanation consists in the transition from laser interference-induced pattern to so-called light-induced periodical surface (LIPPS) [45]. This transition means two different types of incident beam interaction - with reflected light from substrate in the case of grating and interference with light scattered along surface in the case of LIPPS formation. When the angle of the laser beam incident is moving away from the surface normal, a so-called Brewster's angle is achieved and light does not penetrate into polymer films. At these conditions (and at greater angle), light is predominantly reflected or scattered/transmitted along the sample surface. In this case, interaction of incident light with scattered light takes place and LIPPS formation occurs. So, both explanations are possible, and further experiments are in progress to choose the right explanation.We also studied the grating creation based on the initial PMMA-FR thickness. The observed results are given in Figure 8. As can be expected, the grating amplitude is an increasing function of the initial polymer thickness. The amplitude achieves a saturated value when the film thickness exceeds 0.75 μm and then it remains constant. The saturation can be due to limited depth of penetration of the laser light. Due to high absorption coefficient of the PMMA-FR film, the penetration of UV light is restricted only to the near-surface layer. For that reason, there is no difference between the films of larger but different thicknesses. Breakpoint at 0.75 μm can be evaluated as a critical point, at which the amount of transmitted energy is insufficient to induce polymer ablation. Like the previous case, an alternative explanation can be that LIPPS-induced pattern transits into interference-induced pattern. When the polymer thickness is small, light can penetrate through the polymer film twice - to the substrate and from the substrate. So, interference between incident and reflected light is dominant. Increasing the polymer thickness leads to attenuation of light through film thickness. In this case, the interference between scattered light along the surface and the incident light seems to be more possible - LIPPS formation occurs.Ultra-thin polymer films were studied separately, and related AFM scans are presented in Figure 9. The thickness of the polymer film is smaller than the applied wavelength in this case. In this case, a modification of the surface was observed, but the creation of regular pattern was not confirmed. The modified surface rather represents a system of random distributed holes. When the film thickness is close to laser wavelength, the prepared structure becomes more regular and comprises a system of spots. With further increase in film thickness, formation of regular grating pattern occurs. The possible explanation also is that LIPPS interferes with pattern transition: less regular structures in the case of thin films, where interference of reflected and incident beams occurs, and more regular for thick films, where LIPPS structures can be expected.

**Figure 10:**
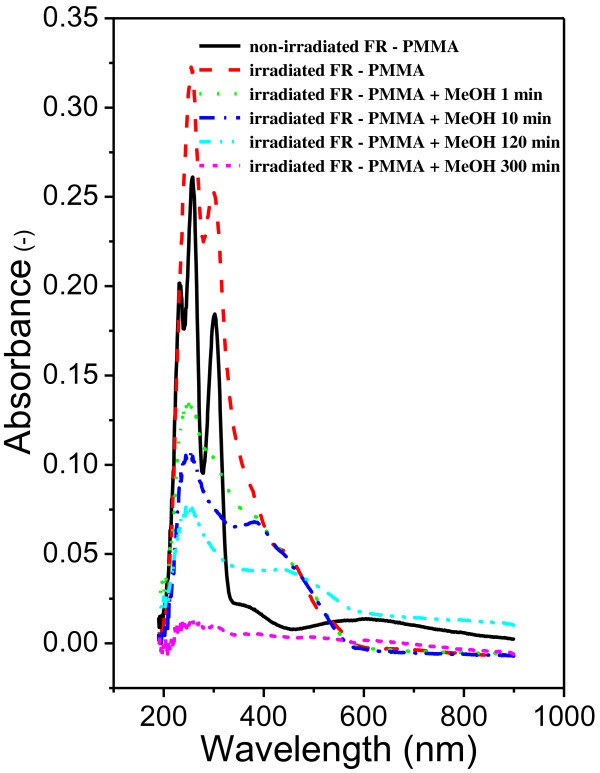
**Absorption spectra of the PMMA-FR films.** Non-irradiated, after irradiation, and after different times (1 to 130 min) of FR extraction in methanol.

**Figure 11:**
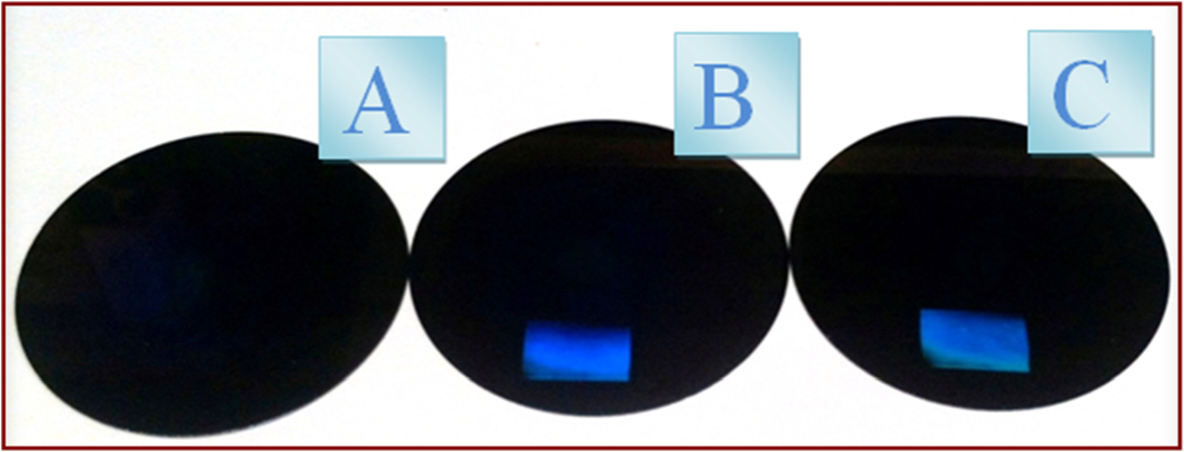
**Photos of the PMMA-FR films. (A)** Pristine, **(B)** treated with laser (with 50 pulses and 12 mJ cm^−2^), and **(C)** soaked in methanol for 30 min.

**Figure 12:**
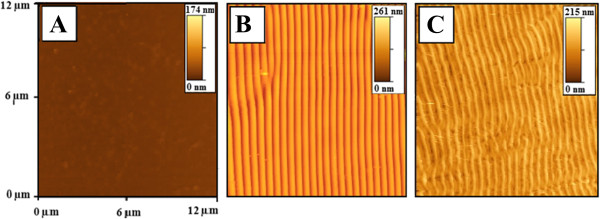
**AFM images of the PMMA-FR films. (A)** Pristine, **(B)** treated with laser (with 50 pulses and 12 mJ cm^−2^), and **(C)** soaked in methanol for 30 min.

Ablation of weakly absorbing polymers can be initiated either by application of high laser energy or by addition of dopant as in our case. However, the presence of dopant can significantly change the properties of a polymer. Doped polymers often exhibit worse thermal and mechanical properties. The tendency of dopants to aggregate and out-diffuse from polymer matrix causes significant decrease of material long-term stability and eliminate practical application of doped polymers [46,47]. For that reason, the FR dopant was removed from the polymer by soaking the polymer in methanol after laser modification. FR is well soluble but PMMA is completely insoluble in methanol. The presence of FR was estimated from UV-vis absorption measurement. Figure 10 gives the optical spectra of the PMMA-FR thin films measured after deposition, laser patterning, and at different times of FR soaking in methanol. Typical FR peaks at 230 to 300 nm indicates its residual amount after extraction. As can be expected, FR diffuses through the polymer matrix, and after 300 min of film soaking, ‘FR-free’ polymer film was obtained.Figure 11 gives the optical images of pristine, laser-modified, and FR-free PMMA films. The diffraction pattern is well visible after laser modification. After methanol soaking, light diffraction becomes less noticeable. However, polymer films remain optically active. Finally, Figure 12 represents the AFM images of the surface pattern of the previous case. The best light diffraction noticeable in the case of patterned PMMA-FR film is clearly visible and the surface structure is close to the ideal one. After soaking in methanol, the regularity of the prepared gratings decreases. The surface pattern is not as good as that in the previous case. On the other hand, the grating structure remains well visible in both optical and surface morphology measurements.

## Conclusions

The grating formation by laser light diffraction on the surface of a PMMA-FR film under extremely mild conditions was described. The grating pattern was observed already after irradiation with 15 pulses of light from KrF laser and 12 mJ cm^−2^ fluence. Creation of surface ordered structures begins at hot places and tends to form concentric semi-circles around. When the number of hot places is high and they are located closely to each other, created structures overlap during further irradiation stages and form highly regular grating pattern. The mechanism of surface ordered structure formation was attributed to ablation, which is more pronounced in the places of higher light intensity. The smoothness of substrate and its high refractive index play a key role in the quality of the prepared gratings. When atomically ‘flat’ Si substrates were used, nearly ideal grating structures are obtained. In some experiments, FR dopant was removed after laser patterning from bulk polymer by soaking the film in methanol. After dopant removal, the grating structures on dopant-free PMMA were obtained. After dopant extraction, the quality of surface ordered structures was partially degraded, but it still remains optically active. It can be assumed that the proposed method of grating creation could find applications in the preparation of optical components, including Bragg grating, wavelength splitter, or active substrates for surface enhancement of luminescence or Raman signals [48].

## Competing interests

The authors declare that they have no competing interests.

## Authors’ contributions

YK carried out the polymer laser modification, dopant extraction, and optical measurements. OL conceived of the study, participated in its design and coordination, and wrote the manuscript. PS carried out the surface analysis and helped in the interpretation of results. RE participated in the laser treatment of the polymer films. VS participated in the design of the study and provided scientific advice. All authors read and approved the final manuscript.
